# Occupational Role Stress and Associated Factors Among Nurses in Southeast Iran

**DOI:** 10.1155/nrp/6651221

**Published:** 2025-02-27

**Authors:** Raheleh Hashemi Habybabady, Hassan Okati-Aliabad, Mahdi Mohammadi

**Affiliations:** Health Promotion Research Center, Zahedan University of Medical Sciences, Zahedan, Iran

**Keywords:** nurses, occupational roles, occupational stress

## Abstract

Occupational role stress is one of the important factors affecting nurses' occupational stress and quality of life (QOL). This study aimed to explore occupational role stress and associated factors among nurses in Zahedan, Southeast Iran. This cross-sectional study was conducted from April to June 2023. The study sample consisted of 260 nurses from hospitals affiliated with Zahedan University of Medical Sciences selected through a multistage sampling method. Data on nurses' occupational role stress, sociodemographic characteristics, lifestyle, sleep quality, and work-related factors were collected. A general linear regression model was applied to determine the factors associated with occupational role stress. The findings revealed that the mean total occupational role stress score was 141.41. Among the occupational role stress subscales, nurses had higher mean scores in role overload, role inadequacy, and responsibility. Total occupational role stress score was significantly higher in university graduate nurses than in those holding a high school diploma (*p*=0.005). Moreover, occupational role stress was significantly higher in nurses who slept for 4–6 h (*p*=0.011) and more than 8 h (*p*=0.017) compared to those who slept for 7–8 h a day. In addition, occupational role stress was also significantly higher in nurses with poor sleep quality (*p*=0.03). In addition, nurses who worked more than 8 h a day reported significantly higher occupational role stress (*p*=0.008). There was no statistically significant association between occupational role stress with age, gender, marital status, BMI, exercise, having a second job, shift work, ward assignment, and work experience. The findings emphasize the necessity of tailored interventions for managing occupational role stress in nurses with higher education and longer work hours. Improving work conditions, along with promoting healthy sleep habits, is a crucial step in reducing occupational role stress.

## 1. Introduction

Occupational stress remains a pervasive issue within the nursing profession, negatively affecting the QOL of nurses and the quality of patient care they deliver [1, 2]. Evidence shows that role stress is a major factor in determining job satisfaction and a primary reason for nurses leaving the workforce [3, 4]. A survey on the effects of workload and job stress on shift work disorders among nurses shows that workload influences job stress, mental and physical health, sleep quality, and personal/family problems, while job stress affects mental health, physical health, sleep quality, and personal/family problems [5].

Occupational role stress refers to the stress experienced by individuals due to the demands and expectations associated with their professional roles [6]. Occupational role stress, such as role overload, role insufficiency, role boundary, and responsibility, have been well documented to be associated with occupational stress in nurses and doctors, even when considering other factors [7, 8]. Moreover, evidence indicated that among occupational role stress, role overload, role insufficiency, and physical environment are significant predictors of the QOL for medical professionals, including nurses [9, 10]. In addition, occupational roles as work stressors are primary risk factors for the three dimensions of burnout in medical professionals. Work stressors directly impact burnout and also indirectly affect it through personal strain and coping resources [11, 12]. Also, role stressors can act as predictors of nursing staff turnover intention [13]. In addition, the predictive power of role overload, role insufficiency, and role boundaries in predicting depressive symptoms in nurses has been proven [14].

An investigation of occupational role stress and related dyslipidemia among medical workers in China showed that the exposure to occupational role stress was significantly associated with dyslipidemia. Low-density lipoprotein cholesterol (LDL-C) and total cholesterol (TC) levels were positively correlated with occupational role stress, while high-density lipoprotein cholesterol (HDL-C levels were negatively correlated after adjustments for various factors [15].

Many previous studies have explored occupational stress and related factors in nurses, sometimes yielding contradictory results, but few have specifically focused on occupational role stress [1, 16–18]. Therefore, this study aimed to explore occupational role stress and its associated factors among nurses in Zahedan, Southeast Iran. The relationship between occupational role stress and demographic characteristics, lifestyle factors, and work-related factors was investigated in this study.

## 2. Materials and Methods

### 2.1. Study Design and Participants

This is a cross-sectional study conducted to explore occupational role stress and its associated factors among nurses in Southeast Iran. Participants were recruited from four hospitals affiliated with Zahedan University of Medical Sciences in Southeast Iran using a multistage random sampling method. In the first stage, affiliated hospitals were stratified based on the type (general or specialized). A random selection of hospitals was then made from each stratum to ensure diverse representation. In the second stage, wards within the selected hospitals were categorized based on specialty (e.g., internal medicine, surgery, and intensive care). Wards were randomly selected from each hospital to capture a variety of nursing work environments. In the third stage, nurses from the selected wards formed the final sampling unit. A list of eligible nurses was obtained from ward rosters, and participants were randomly selected using a random number generator to ensure fairness and an equal chance of participation.

Nurses working in clinical activities in hospital wards with at least 1 year of work experience who were willing to participate in the study were eligible. Nurses with a history of mental health conditions, and those who were treated with sleeping pills were excluded from the study.

To determine the sample size, the standard deviation of the total occupational role stress score was estimated as 24.5 based on a pilot study involving 50 participants. Using a Type I error of 0.05 and an estimation error of 3, the sample size was calculated to be 257. A total of 260 nurses ultimately participated in the study.

Ethical approval was obtained from the Ethics Committee of Zahedan University of Medical Sciences (Approval Number: IR.ZAUMS.REC.1400.261). All participants provided informed consent, ensuring voluntary participation, confidentiality, and anonymity.

### 2.2. Data Collection

Data were gathered between April and June 2023. The respondents were from different wards of the hospitals. The data collection tool was a self-administered questionnaire that included sections on sociodemographic characteristics, lifestyle, work characteristics, sleep quality, and occupational role stress. The sociodemographic section covered age, gender, marital status, and education level. Lifestyle factors included BMI, exercise, sleeping hours, and sleep quality. Job characteristics encompassed work experience, daily working hours, having a second job, and shift work.

The Persian version of the Occupational Role Questionnaire (ORQ), validated in the prior research [19], was used to assess participants' occupational role stress. ORQ is a subsection of the Occupational Stress Inventory-Revised Edition (OSI-R) [6]. The OSI-R consists of three questionnaires: the ORQ, the Personal Strain Questionnaire (PSQ), and the Personal Resource Questionnaire (PRQ). In the current study, only the ORQ was used to evaluate occupational role stress as work stressors. The ORQ includes six aspects: role overload, role ambiguity, role insufficiency, role boundary, responsibility, and physical environment. Each aspect contains 10 items scored from 1 to 5. The scores for each aspect were summed to provide a total score for that specific area, with higher scores indicating greater levels of occupational role stress. The overall ORQ score was calculated by summing the scores of the five aspects. Consequently, a higher ORQ score reflects greater overall occupational role stress. Previous studies have showed that the “physical environment” component is not related to the hospital environment [8, 20]. Therefore, the physical environment was excluded from this study.

We determined the body mass index (BMI) of the participants by dividing their weight in kilograms by their height in meter squared (m^2^). Participants were then categorized as follows: underweight with a BMI of less than 18.5 kg/m^2^, normal weight with a BMI of 18.5–24.9 kg/m^2^, overweight with a BMI of 25–29.9 kg/m^2^, and obese with a BMI of over 30 kg/m^2^.

The Persian version of the Pittsburgh Sleep Quality Index (PSQI), validated in the prior research [21], was used to evaluate overall sleep quality. The PSQI, a widely recognized tool, measures sleep quality over 1 month and is particularly suited for evaluating sleep quality among nursing staff [22]. It consists of 19 self-rated questions, divided into seven components: sleep duration, sleep disturbances, subjective sleep quality, sleep latency, habitual sleep efficiency, daytime dysfunction, and use of sleep medication. Each component is rated on a scale from 0 (*no difficulty*) to 3 (*severe difficulty*). The scores for the seven components are then summed to produce a global PSQI score, ranging from 0 to 21, with higher scores indicating poorer sleep quality. A global score greater than 5 typically signifies poor sleep quality [23].

The instruments utilized in this study were pretested on a sample of 50 individuals to ensure their appropriateness and relevance. Furthermore, the reliability of the questionnaire was confirmed by calculating Cronbach's alpha (≥ 0.70), indicating satisfactory internal consistency of the measures.

### 2.3. Statistical Analysis

Quantitative and qualitative variables were described as mean values ± standard deviation and frequency distribution, respectively. The Shapiro–Wilk test was employed to assess the normality of the data, while Levene's test was used to evaluate the homogeneity of variances. The correlation between total occupational role stress and its subscales was assessed using Pearson's correlation test. One-factor and multifactor general linear models were used to determine the relationship between independent factors and occupational role stress. Data analysis was performed using SPSS version 24, with a significance level set at 0.05.

## 3. Results

A total of 260 nurses with a mean age of 32.66 ± 7.80 years participated in this study. Most of the nurses were female (64.2%), married (70%), and university graduates (88.5%). About half of the nurses had a normal weight and engaged in regular exercise. Most nurses reported up to 10 years of work experience (72.4%), shift work (85%), 7-8 h of sleep (54.2%), and 6–8 working hours per day (82.3%) (Table 1).

The mean score of total occupational role stress was 141.41 ± 17.86 out of 250. Among the occupational role stress subscales, nurses had higher mean scores in role overload, role inadequacy, and responsibility. All occupational role stress subscales were significantly correlated with total occupational role stress (Table 2).

Total occupational role stress was not significantly related to age (*p*=0.105), gender (*p*=0.768), or marital status (*p*=0.628). However, it was significantly higher in university graduate nurses than in those holding a high school diploma (*p*=0.005) (Table 3).

There was no significant relationship between total occupational role stress and BMI (*p*=0.716) or exercise (*p*=0.643). However, occupational role stress was significantly higher in nurses who slept for 4–6 h (*p*=0.011) and more than 8 h (*p*=0.017) compared to those who slept for 7–8 h a day. Occupational role stress was also significantly higher in nurses with poor sleep quality (*p*=0.03) (Table 4).

Occupational role stress was not significantly related to having a second job (*p*=0.538), shift work (*p*=0.931), ward assignment (*p*=0.577), or work experience (*p*=0.082). However, nurses who worked more than 8 h a day reported significantly higher occupational role stress (*p*=0.008) (Table 5).

## 4. Discussion

The findings of this study revealed that the mean score of occupational role stress among nurses was 141.41 out of a possible 250, indicating a significant level of occupational role stress within this population. This score reflects the cumulative impact of various stressors inherent to nursing roles. In the present study, the mean scores of all ORQ subscales among Iranian nurses were higher than those of Chinese nurses [24, 25]. These findings indicate that Iranian nurses experience more occupational stressors than their Chinese counterparts. Among the occupational role subscales assessed, nurses demonstrated higher mean scores specifically in role overload, role insufficiency, and responsibility. A previous study confirmed that these dimensions were prominent sources of stress among nurses [20].

This study showed that among the demographic factors, age, gender, and marital status were not significantly related to occupational role stress. It indicates that occupational stressors affect nurses regardless of their age, gender, or marital status. This finding aligns with some existing literature studies. A previous study in Iran showed no significant relationship between the overall score of occupational role stress, including each aspect, and the marital status of nurses [26]. Another study conducted during the COVID-19 pandemic in Iran revealed that demographic factors, including age and marital status, were not associated with occupational stress among nurses [27]. Bahrami et al. found no significant relationship between occupational role stress and gender, age, or marital status [28]. However, in the current study, nurses with a university degree reported higher levels of occupational role stress compared to those with a high school diploma. This may be attributed to their greater awareness of the complexities and demands of their roles. In line with the present study, a previous study in Iran showed that the level of occupational stress increases at higher levels of education [16].

Among lifestyle factors, this study revealed no significant relationship between BMI or exercise with total occupational role stress. The lack of a significant relationship between total occupational role stress and BMI or exercise may be due to the ineffectiveness of some exercise interventions in reducing stress as general physical activity programs at work have not had significant stress-reducing effects among health personnel, including nurses [29, 30]. In contrast, sleep duration and quality showed significant associations with occupational role stress. The literature suggests that insomnia and short sleep duration are associated with an increased risk of poor workability, individually and in combination [31]. Therefore, work-related insomnia and short sleep duration may make it more difficult for nurses to cope with the demands of their roles.

This study demonstrates that among work-related factors, there was no significant relationship between total occupational role stress with having a second job, shift work, ward assignment, or work experience. However, a significant relationship was found with the number of hours worked per day, with nurses working more than 8 h a day reporting significantly higher levels of occupational role stress. In line with this study, the research on occupational role stress among Iranian CCU nurses showed no statistically significant difference between occupational role stress and work experience [19]. Furthermore, a previous study in India found no significant relationship between occupational role stress aspects and working in different wards, indicating that a positive work environment can reduce stress even in more intense hospital wards [17].

In the current study, nurses working more than 8 h a day reported significantly higher occupational role stress. This finding is consistent with the extensive research linking long working hours with increased stress. A study in Ethiopia revealed that nurses working more than 8 h per day were twice as stressed as those working 8 h or less per day [32]. In a study among Iranian nurses, increased work hours were identified as a risk factor for occupational stress [33].

## 5. Study Limitation

This study has several limitations that must be taken into account when interpreting the findings. Firstly, the cross-sectional design restricts the ability to determine causal relationships between occupational role stress and the associated factors. Longitudinal studies are needed to better understand the temporal relationships and causality. Secondly, the data were self-reported, which may introduce response bias and affect the accuracy of the information provided. Future research should address these limitations by using a longitudinal design and incorporating objective measures to enhance the robustness of the findings.

## 6. Conclusion

This study's findings reveal that occupational role stress is a significant issue affecting nurses, with higher levels observed in those with advanced education and extended work hours. Factors such as age, gender, marital status, BMI, and exercise were not significantly related to occupational role stress. However, poor sleep quality and extreme sleep durations (both short and long) were associated with higher stress levels. These results highlight the critical need for targeted interventions to manage occupational role stress, particularly for nurses with higher education and those working extended hours. Improving work conditions and promoting healthy sleep habits are essential components of occupational role stress reduction programs.

## Figures and Tables

**Table 1:** Frequency distribution of sociodemographic characteristics, lifestyle, and work-related factors.

Variable	*N* (%)
*Age (year)*	
≤ 25	39 (15.0)
26–35	145 (55.8)
36–45	56 (21.5)
> 45	20 (7.7)

*Sex*	
Male	93 (35.8)
Female	167 (64.2)

*Education*	
High school diploma	30 (11.5)
Associate/BSc degree	221 (85.0)
MSc degree	9 (3.5)

*Marital status*	
Single	78 (30.0)
Married	182 (70.0)

*BMI*	
Underweight	16 (6.2)
Normal	133 (51.2)
Overweight	96 (36.9)
Obese	15 (5.8)

*Exercise*	
No	130 (50.0)
Yes	130 (50.0)

*Sleeping hours*	
4–6	92 (35.4)
7–8	141 (54.2)
> 8	27 (10.4)

*Work experience (year)*	
1	41 (15.8)
2–5	86 (33.1)
6–10	61 (23.5)
11–15	36 (13.8)
> 15	36 (13.8)

*Ward*	
Emergency/CCU/ICU/NICU	129 (49.6)
Others	131 (50.4)

*Working hours*	
6–8	214 (82.3)
> 8	46 (17.7)

*Second job*	
No	225 (86.5)
Yes	35 (13.5)

*Shift work*	
No	39 (15.0)
Yes	221 (85.0)

**Table 2:** Correlation between total occupational role stress and its subscales.

Occupational role stress subscales	Mean ± SD	Maximum possible score	Correlation coefficient (*p* value)
Role overload	29.58 ± 6.27	10–50	*r* = 0.612 (*p* < 0.001)
Role insufficiency	28.89 ± 6.14	10–50	*r* = 0.610 (*p* < 0.001)
Role ambiguity	26.88 ± 6.20	10–50	*r* = 0.456 (*p* < 0.001)
Role boundary	27.32 ± 5.47	10–50	*r* = 0.637 (*p* < 0.001)
Responsibility	28.73 ± 5.26	10–50	*r* = 0.523 (*p* < 0.001)
Total occupational role stress	141.41 ± 17.86	50–250	

**Table 3:** Relationship between sociodemographic characteristics and total occupational role stress.

Variable	One-factor model		Multifactor model	
	Mean ± SD	*p* value	*B* ± SE	*p* value
Age (year)		0.105		
≤ 25	142.82 ± 16.29			
26–35	140.98 ± 17.47			
36–45	144.45 ± 19.36			
> 45	133.25 ± 17.81			
Gender		0.768		
Male	140.97 ± 18.58			
Female	141.65 ± 17.50			
Education		0.005		0.005
High school diploma	132.77 ± 16.80		−9.77 ± 3.42	0.005
Associate/BSc/MSc degree	142.53 ± 17.72		Ref.	
Marital status		0.628		
Single	142.23 ± 16.17			
Married	141.05 ± 18.57			

**Table 4:** Relationship between lifestyle factors and total occupational role stress.

Variable	One-factor model		Multifactor model	
	Mean ± SD	*p* value	*B* ± SE	*p* value
BMI		0.716		
Underweight	138.50 ± 15.15			
Normal weight	142.43 ± 18.24			
Overweight	140.96 ± 18.32			
Obese	138.33 ± 14.57			
Exercise		0.643		
No	140.89 ± 18.44			
Sometimes/always	141.92 ± 17.32			
Sleeping hours		0.002		0.008
4–6	144.87 ± 19.65		6.04 ± 2.36	0.011
7–8	137.96 ± 16.97		Ref.	
> 8	147.59 ± 11.34		8.82 ± 3.67	0.017
Sleep quality		0.007		0.030
Good	136.40 ± 17.02		−5.45 ± 2.49	0.030
Poor	143.18 ± 17.85		Ref.	

**Table 5:** Relationship between work-related factors and total occupational role stress.

Variable	One-factor model		Multifactor model	
	Mean ± SD	*p* value	*B* ± SE	*p* value
Working hours		0.008		0.008
≤ 8	140.04 ± 18.27		−7.72 ± 2.87	0.008
> 8	147.76 ± 14.37		Ref.	
Second job		0.538		
No	141.14 ± 17.11			
Yes	143.14 ± 22.35			
Shift work		0.931		
No	141.18 ± 15.73			
Yes	141.45 ± 18.25			
Ward		0.737		
Emergency/CCU/ICU/NICU	141.78 ± 19.20			
Others	141.04 ± 16.51			
Work experience (years)		0.082		
1	137.22 ± 16.21			
2–5	143.06 ± 17.35			
6–10	145.48 ± 18.44			
11–15	138.69 ± 18.35			
> 15	138.06 ± 18.30			

## Data Availability

The data that support the findings of this study are available from the corresponding author upon reasonable request.
